# Integrated multi-omics and bioinformatic methods to reveal the mechanisms of sinomenine against diabetic nephropathy

**DOI:** 10.1186/s12906-023-04119-0

**Published:** 2023-08-14

**Authors:** Yan Li, Lei Wang, Jimin Zhang, Bojun Xu, Huakui Zhan

**Affiliations:** 1 Department of Rheumatology and Clinical Immunology, The First Affiliated Hospital of Xiamen University, Xiamen, 117892 Fujian China; 2 Xiamen Municipal Clinical Research Center for Immune Diseases, Xiamen, 361000 XM China; 3 Xiamen Key Laboratory of Rheumatology and Clinical Immunology, Xiamen University, Xiamen, 12466 Fujian China; 4 Hospital of Chengdu University of Traditional Chinese Medicine, Chengdu, 610072 Sichuan China; 5 Key Laboratory of Chinese Internal Medicine of Ministry of Education and Dongzhimen Hospital, Beijing University of Chinese Medicine, Beijing, 100700 China

**Keywords:** Diabetic nephropathy, Sinomenine, Metabonomics, Transcriptomics, Network pharmacology

## Abstract

**Objectives:**

Diabetic Nephropathy (DN) is a serious complication of diabetes, the diagnosis and treatment of DN is still limited. Sinomenine (SIN) is an active extract of herbal medicine and has been applied into the therapy of DN.

**Methods:**

In the part of bioinformatic analyses, network pharmacology and molecular docking analyses were conducted to predict the important pathway of SIN treatment for DN. In-vivo study, DN rats were randomized to be treated with vehicle or SIN (20 mg/kg or 40 mg/kg) daily by gavage for 8 weeks. Then, the pharmacological effect of SIN on DN and the potential mechanisms were also evaluated by 24 h albuminuria, histopathological examination, transcriptomics, and metabolomics.

**Results:**

Firstly, network pharmacology and molecular docking were performed to show that SIN might improve DN via AGEs/RAGE, IL-17, JAK, TNF pathways. Urine biochemical parameters showed that SIN treatment could significantly reduce 24 h albuminuria of DN rats. Transcriptomics analysis found SIN could affect DN progression via inflammation and EMT pathways. Metabolic pathway analysis found SIN would mainly involve in arginine biosynthesis, linoleic acid metabolism, arachidonic acid metabolism, and glycerophospholipid metabolism to affect DN development.

**Conclusions:**

We confirmed that SIN could inhibit the progression of DN via affecting multiple genes and metabolites related pathways.

## Introduction

Diabetic Nephropathy (DN) refers to kidney damage caused by diabetes, which is a common and serious complication of diabetes leading to the development of end-stage kidney disease [1]. Epidemiological studies show that diabetes currently influences more than 425 million people around the world and the incidence was estimated to rise to about 592 million by 2035 worldwide [2, 3]. About 30-40% of diabetic patients will develop DN, and about one third of patients will further develop end-stage renal disease, putting a heavy economic burden on the society [1]. The pathological characteristics of DN are usually featured as thickened base membrane, extracellular matrix deposition, glomerular hypertrophy, sclerosis, and interstitial fibrosis [4].

For decades, there is nearly no effective treatment for DN though the pathological mechanisms of DN have been deeply studied [5], and DN patients mainly just be suggested to control the blood glucose and blood pressure [6]. Currently, Sodium-glucose co-transporter 2 (SGLT2) inhibitors are beneficial to many DN pathophysiological abnormalities, however it needs multiple agents used in combination and has many side effects [7]. Sinomenine (SIN), an active compound extracted from Chinese herbal medicine and widely applied in treating many autoimmune diseases, and recent study displayed that SIN has renal protective properties [8]. Zhu et al. found that SIN could involve in protecting nephrocytes and decreasing renal tissue injury through oxidative stress inhibition, renal cell apoptosis and fibrosis reducing, modulating the JAK2/STAT3/SOCS1 pathway in DN rats [9]. Moreover, Yin et al. reported that SIN could effectively improve hyperglycemia-disrupted renal endothelial barrier function through suppressing the RhoA/ROCK signaling via inhibiting ROS [10].

**Fig. 1:**
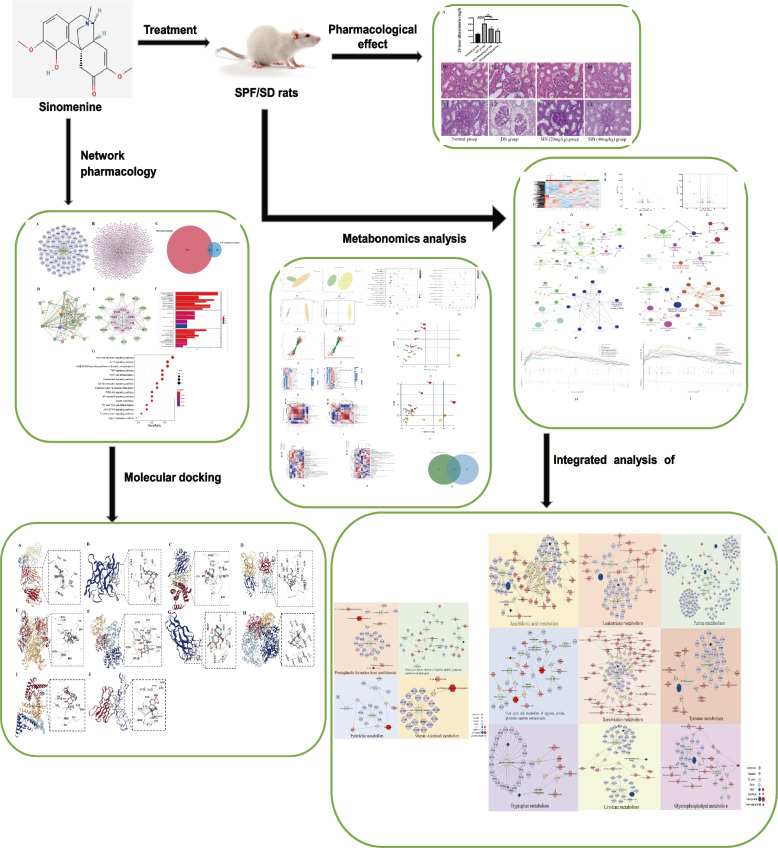
A flow chart of the whole study

Network pharmacology is becoming a common methodology to predict the potential targets of a drug or compound to treat the disease [11]. In addition, molecular docking can calculate the ligand-receptor binding energy in order to evaluate the level of correlation between ligand and receptor [12]. Metabolomics analysis is a rapidly emerging field in systems biology, which can observe metabolic changes and enable us to understand the potential mechanisms in the development and progression of the disease [13]. Transcriptomics study relied on high-throughput sequencing provides an approach to rapidly determine the changes in mRNA level after drug administration, assisting us to figure out the mechanisms of the drugs [14]. In our study, we combined network pharmacology, histology, 24 h albuminuria, molecular docking, and multi-omics to indicate the potential molecular mechanisms of which SIN involves in renal protection (Fig. 1).

## Methods and materials

### Reagents, chemicals and kits

Streptozotocin (STZ) was purchased from Sigma-Aldrich. Sinomenine (purity > 98%) was purchased from Shanghai Roche Pharmaceutical Co., Ltd. (Shanghai, China). Urinary protein concentration was estimated with a protein assay kit (Jiancheng Bio, Nanjing, China). TRIzol® Reagent was purchased from Invitrogen. Hematoxylin, eosin and periodic acid-Schiff were from Wuhan servicebio technology CO., Ltd. (Wuhan, China).

### Network pharmacology analysis

The potential targets of DN were searched via inputting the keyword of “diabetic nephropathy” in Genecards (https://www.genecards.org/), a database with data about genomics, proteomics, and transcriptomics [15]. Similarly, DisGeNET database (https://www.disgenet.org/home/) was also applied to collect the potential diasease-related targets [16].

The molecular targets of SIN were screened via inputting the keywords “sinomenine” from TCMSP (http://lsp.nwu.edu.cn/tcmsp.php) [17], Swiss Target Prediction (http://www.swisstargetprediction.ch.) [18], and HERB database (http://drug.ac.cn/) [19]. Then, these proteins were standardized via applying UniProtKB database (http://www.uniprot.org/) [20].

The screened SIN and DN targets were inputted into Funrich software for getting the overlapping targets [21]. Then, A protein–protein interaction (PPI) network was constructed via applying STRING (https://string-db.org/) [22] and Cytoscape software [23]. Hub genes were selected by using CytoHubba plug-in of Cytoscape [24].

The “ClusterProfiler” R package was applied to conduct the Gene Ontology (GO) and Kyoto Encyclopedia of Genes and Genomes (KEGG) enrichment analysis, P < 0.05 was applied as a screening threshold. GO is a comprehensive platform for classifying gene functions, covering biological process (BP), molecular function (MF), and cell component (CC) [25]. KEGG (http://www.kegg.jp/) is an widely-used encyclopedia to identify significantly enriched biological pathways of targets [26].

### Molecular docking

We applied CB-Dock (http://cao.labshare.cn/cb-dock/) for molecular docking to predict the binding ability of SIN and core targets. CB-Dock is a molecular docking tool based on AutoDock Vina for calculation, which can automatically identify the site where the ligand binds to the receptor, and analyze the center position and size of the binding site [27]. Protein file and ligand file were obtained from PDB (https://www.rcsb.org/) [28] and Pubchem (https://pubchem.ncbi.nlm.nih.gov/) [29], respectively.

### Animals and preparation of DN animal model

The male inbred SD rats, 6–8 weeks old, weighing 200 ± 20 g, were obtained from Beijing Vital River Laboratory Animal Technology Co., Ltd. (Beijing, China) (Animal Certificate No: SCXK [Jing] 2021-0006). After adaptive feeding, all rats were randomly distributed into four groups (n = 6 per group): normal group; DN group; SIN (20 mg/kg) administration group; SIN (40 mg/kg) administration group [30].

We used 0.1 M citrate buffer (pH 4.5) to prepare streptozotocin and injected the freshly prepared STZ to overnight fasting rats at a 45 mg/kg dose. After one week, rats with a blood glucose level over 16.7 mmol/L were selected in subsequent experiments. In normal group, equal volume of sodium citrate buffer was injected. The rats in normal group were fed in normal diet, while the remaining groups were administered with high-glucose and high-fat diet. In addition, SIN (20 mg/kg) administration group was treated with SIN (20 mg/kg) per day and SIN (40 mg/kg) administration group was treated with SIN (40 mg/kg) per day. The animal experiment was approved by animal experiment center (IACUC-P2021-077). Urinary protein concentration was estimated with a protein assay kit.

### Histology

After removing the kidneys of rats, we fixed them in 4% paraformaldehyde in 0.1 M PBS, embedded in paraffin, and cut into 5-mm sections. In order to discover the morphological changes of renal tissue, sections were stained with hematoxylin and eosin (H&E) and periodic acid-Schiff (PAS). These renal sections were observed by applying light microscopy, then digital images were obtained and analyzed.

### Transcriptomics

Total RNA was extracted from renal tissues of each group using TRIzol® Reagent and the mRNA was enriched by Oligo(dT). Then the mRNA was scattered by fragmentation buffer. Eukaryotic mRNA sequencing was performed using the Illumina Novaseq 6000 (Illumina, San Diego, USA) sequencing platform. The raw reads were subjected to adapter trimming and quality controlled by SeqPrep (https://github.com/jstjohn/SeqPrep) and Sickle (https://github.com/najoshi/sickle). The high-quality clean reads were aligned to the Rat genome using TopHat (http://tophat.cbcb.umd.edu/, version2.1.1). Rat genome sequence and gene annotation were obtained from the Rnor_6.0 Website (http://asia.ensembl.org/Rattus_norvegicus/Info/Index). EdgeR package of R software was applied for differential expression analysis for identifying differential expression genes (DEGs) between DN and SIN-treated group, the expression levels of every transcript was estimated based on the fragments per kilobase of exon per million mapped reads (FPKM) method. The databases including NR (Version 2019.6.26), Swiss-prot (Version 2019.7.1), Pfam (Version v32.0), GO (Version 2019.7.1) and KEGG (Version 2020.03) were applied to data process. “DESeq” R package was applied to calculate the p-adjust value and log2FC. If the p-adjust < 0.05 & |log2FC| >= 1, it was accepted to be a significantly different expression level.

### Metabolomic profiling

LC-MS/MS analyses were conducted via using a UHPLC -Q Exactive HF-X system (Thermo, USA) with a HSS T3 column (1.8 μm; 2.1 × 100 mm). A quality control sample (QC) prepared by mixing equal volume of each sample was disposed and tested in the same manner as the analytic sample. The mass spectrometric data was obtained by applying a Thermo UHPLC -Q Exactive HF-X Mass Spectrometer equipped with an electrospray ionization (ESI) source operating in either positive or negative ion mode. The raw data were inputted into the Progenesis QI 2.3 (Nonlinear Dynamics, Waters, USA) to detect and align peaks. Target metabolites were evaluated via using accurate mass, MS/MS fragments spectra and isotope ratio difference with screening in biochemical databases such as Human metabolome database (HMDB) (http://www.hmdb.ca/) and Metlin database (https://metlin.scripps.edu/). Principle component analysis (PCA) as an unsupervised method was employed to visualize an overview of the metabolic data, general clustering, trends, or outliers. In addition, orthogonal partial least squares discriminate analysis (OPLS-DA) was applied for statistical analysis to identify overall metabolic changes between DN and SIN-treated group. Statistically significant between two groups were picked out with VIP value > 1 as well as *p* value < 0.05. We summarized the Differential metabolites between DN and SIN-treated group and mapped them into their biochemical pathways through metabolic enrichment and pathway analysis according to KEGG database. These metabolites were categorized based on the pathways they involved or the functions they performed. scipy.stats (Python packages) ( https://docs.scipy.org/doc/scipy/) was employed to determine significantly enriched pathway via using Fisher’s exact test.

### Combined metabolomics and transcriptomics analysis

Cytoscape software 3.7.1 (https://js.cytoscape.org/) is a tool for visualizing the biomedical networks including metabolic, gene and other types of interactions. Specifically, Metscape (http://MetScape.ncibi.org) is a Cytoscape plug-in which enables users to build and analyze networks of genes and compounds, identify enriched pathways according to expression profiling data and visualize changes in metabolite data [31]. The data obtained for differentially abundant metabolites and DEGs from rats in the DN, SIN (20 mg/kg) and SIN (40 mg/kg) treatment group were imported into Metscape to obtain a network diagram of gene and metabolic changes to assess the underlying mechanisms of SIN in treating DN.

### Statistical analysis

Statistical differences of biological parameters between SIN-treated groups and DN group were measured by using one-way ANOVA test. All statistical analyses were performed using GraphPad Prism. *P* < 0.05 was accepted to be statistically significant.

## Results

### Network pharmacology prediction

#### Compound-targets network construction

**Fig. 2:**
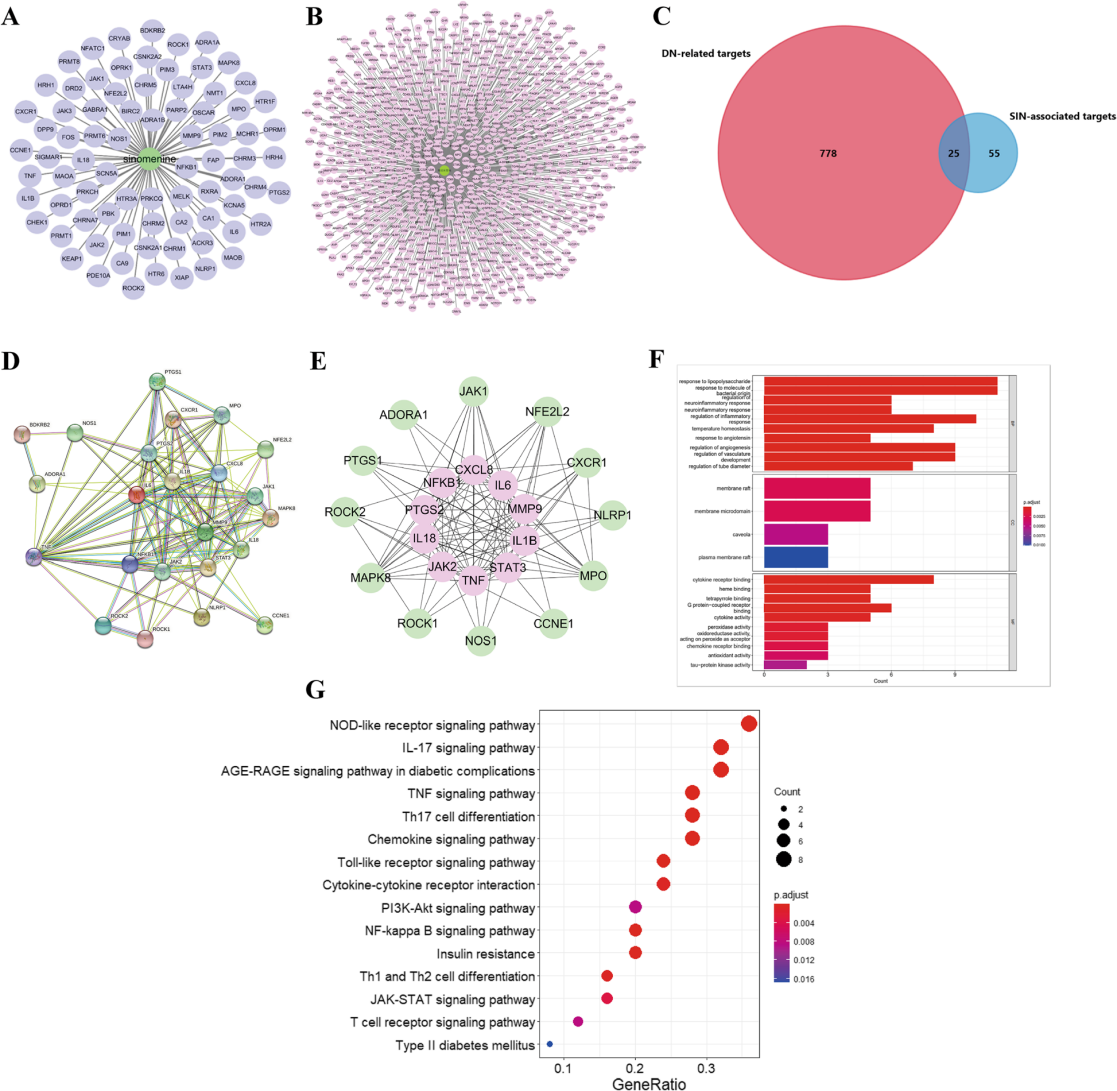
Network pharmacology prediction. Compound-target network of putative targets and SIN (**A**). Potential targets of DN (**B**). Overlapping target genes between DN and SIN (**C**). PPI network of putative targets of SIN and DN (**D**). Determination of the core targets of the PPI network (**E**). Go analysis of the putative targets (**F**). KEGG analysis of the putative targets (**G**)

We constructed a compound-target network by using CytoScape (Fig. 2A) to display the interaction among SIN and its related targets. Candidate targets of SIN were filtered by TCMSP, HERB, and Swiss Target Prediction databases. After UniProt standardizing and removing the duplicate proteins, 80 targets were identified.

#### Determination of DN-related targets

After retrieved the DisGeNET and GeneCards databases, we integrated the results to obtain 803 overlapping DN-associated targets. As shown in Fig. 2B.

#### Drug–disease intersection targets

Venn analysis was conducted via using the 80 targets of SIN and 803 DN-related targets, 25 drug–disease overlapping targets were obtained for later analyses, as shown in Fig. 2C.

#### PPI network analysis

The 25 putative targets were analyzed by using the STRING database to establish a PPI network, as shown in Fig. 2D. The results of STRING analysis were imported into Cytoscape and the core targets were identified by Cytoscape plug-in CytoHubba (Fig. 2E), including CXCL8, IL6, MMP9, IL1B, STAT3, TNF, JAK2, IL18, PTGS2, and NFKB1.

#### GO enrichment and KEGG pathway analyses

After performed GO analysis of 25 putative targets, we found the relevant biological processes which include the response to lipopolysaccharide, regulation of neuroinflammatory response, regulation of inflammatory response, response to angiotensin, regulation of angiogenesis, regulation of vasculature development, and regulation of tube diameter, etc. In addition, we found the cell component included membrane raft, membrane microdomain, caveola, plasma membrane raft. The molecular function consisted of cytokine receptor binding, heme binding, tetrapyrrole binding, G protein − coupled receptor binding, cytokine activity, peroxidase activity, oxidoreductase activity, acting on peroxide as acceptor, chemokine receptor binding, antioxidant activity, and tau − protein kinase activity, etc. (Fig. 2F). KEGG analysis showed the relevant signaling pathways for SIN treating DN include lipid and atherosclerosis, IL-17 signaling pathway, AGE-RAGE signaling pathway in diabetic complications, NOD-like receptor signaling pathway, TNF signaling pathway, JAK-STAT signaling pathway, type II diabetes mellitus, etc. (Fig. 2G).

### Molecular docking study

**Fig. 3:**
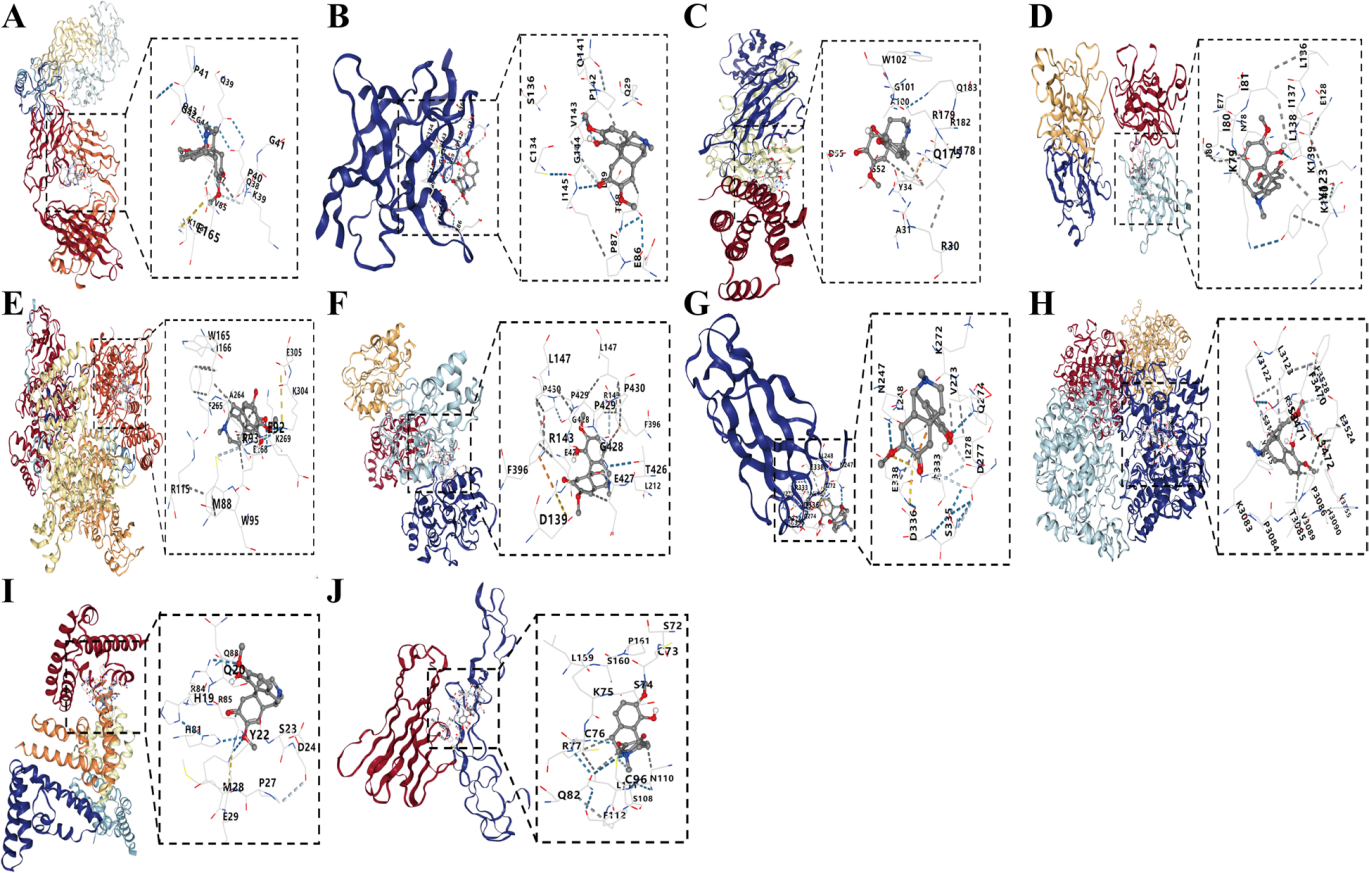
Molecular docking of SIN and core targets. CXCL8 (**A**), IL1B (**B**), IL6 (**C**), IL18 (**D**), JAK2 (**E**), MMP9 (**F**), NFKB1 (**G**), PTGS2 (**H**), STAT3 (**I**), TNF (**J**)

We selected the 10 core targets identified by CytoHubba, including CXCL8, IL6, MMP9, IL1B, STAT3, TNF, JAK2, IL18, PTGS2, and NFKB1. According to the calculation results, CB-Dock output the top 5 conformations with the lowest binding energy value. The binding ability of the ligand and the receptor is related to the Vina score. The lower vina score was always associated with the more efficient and stable the binding energy between ligand and receptor. The results of molecular docking showed that SIN has a good binding ability with the core targets (Table 1). Figure 3 displayed the best docking conformations for the potential targets and SIN.

**Table 1:** The results of SIN-core targets molecular docking

Protein name	Vina	Cavity	Center			Size		
	score	size	x	y	z	x	y	z
TNF	-6.2	1281	38	18	46	27	19	19
IL1B	-5.9	78	3	-14	15	19	19	19
IL6	-7	533	-20	17	27	19	19	19
STAT3	-6.7	1726	106	55	131	19	27	19
MMP9	-7.3	895	19	31	0	19	19	19
CXCL8	-7.1	1468	-24	-21	20	19	35	19
PTGS2	-7.8	4378	62	50	79	26	29	26
JAK2	-7.9	2168	248	-57	37	30	19	19
IL18	-6.9	2706	-7	81	117	31	19	26
NFKB1	-5.7	46	38	32	5	19	19	19

### Effect of SIN on 24 h albuminuria

**Fig. 4:**
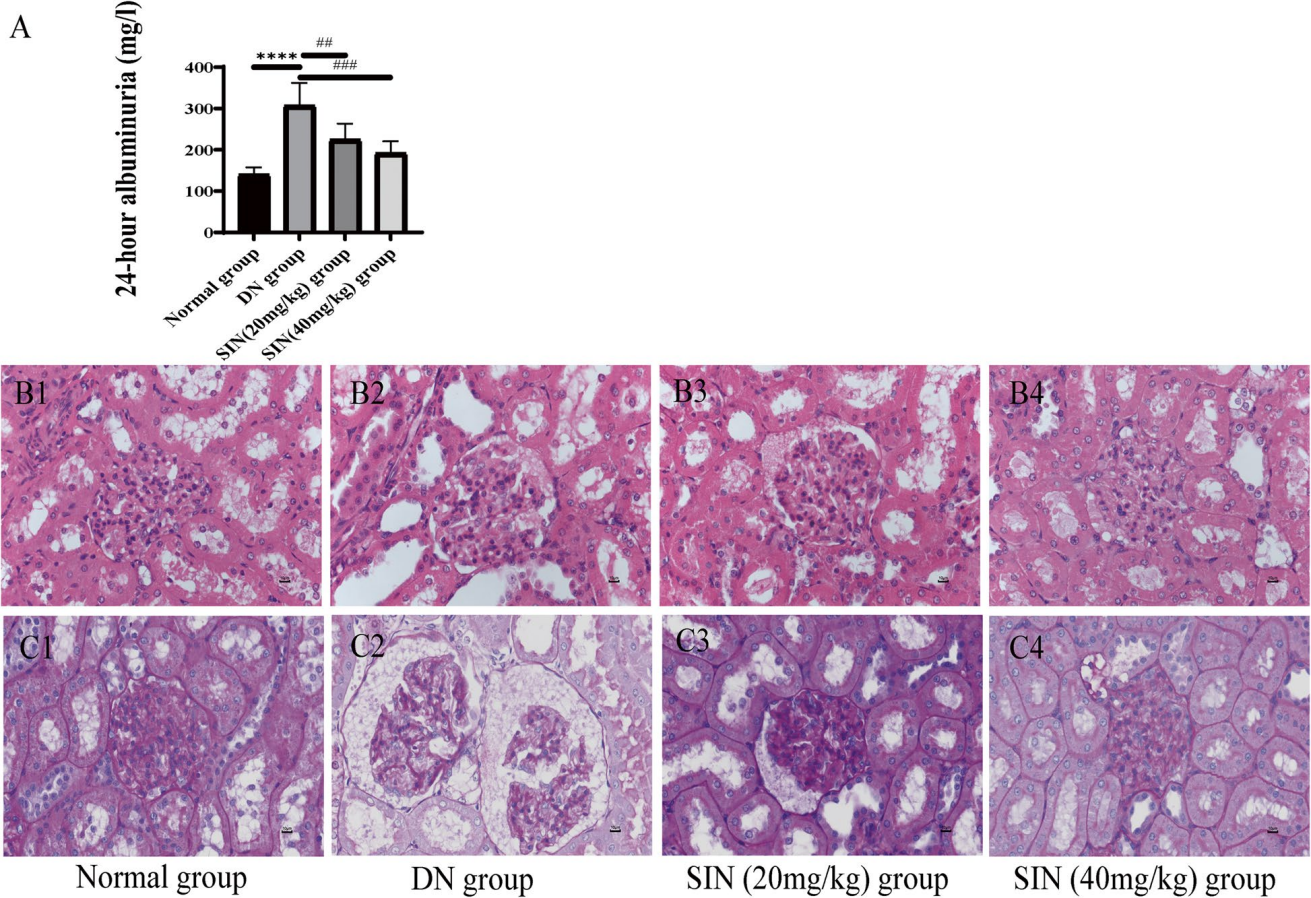
Effects of treatment of SIN at two doses on albuminuria, renal function and nephropathy morphology in DN rats. The treating effect of SIN on albuminuria (**A**). Representative micrographs of kidney sections were conducted in rats. Pathological changes of kidney were evaluated by H&E (**B**), PAS staining (**C**) (scale bar = 10 μm). (****, *p* < 0.0001 vs. normal group; ##, ###, *p* < 0.01, 0.001 vs. DN group, n = 6)

We have measured the levels of urinary albumin to find out the effect of SIN to decrease or normalize those parameters. Detailed results were showed in Fig. 4A. After 8 weeks administration, the 24-hour urinary total protein of rats in SIN (20 mg/kg) group were 223.8 ± 39.59 mg/l versus 306.6 ± 55.45 mg/24 h in DN group (*P* < 0.01), and in SIN (40 mg/kg) group were 191.1 ± 29.40 mg/1 versus 306.6 ± 55.45 mg/l in DN group (*P* < 0.001). The data above indicated that SIN could play a protective role on renal function in DN progression.

### Results of the histological evaluation of kidney

Histological examination of renal tissue was conducted by using H&E, PAS staining as shown in Fig. 4B, C. In the H&E staining, obvious sclerosing glomeruli, vacuolation of tubular epithelial cells, obvious dilation of renal tubules, mesangial expansion, and thickened basement membranes were exhibited in DN group. Compared with the DN group, the basement membrane, glomerular and tubular structure in normal group were complete. After SIN (20 mg/kg) and SIN (40 mg/kg) treatment, the pathological phenomena of DN renal tissues were significantly improved, the atrophic glomeruli were ameliorated, and the tubular dilatation was significantly reduced. In addition, PAS staining displayed the excessive extracellular matrix deposition inside glomeruli and on the basement membrane of the tubule in DN group. Compared with the SIN-treated group, the pathological abnormalities above were obviously improved. These histological results revealed that SIN had considerable therapeutic potential on DN progression.

### Transcriptomic sequencing and data analysis

**Fig. 5:**
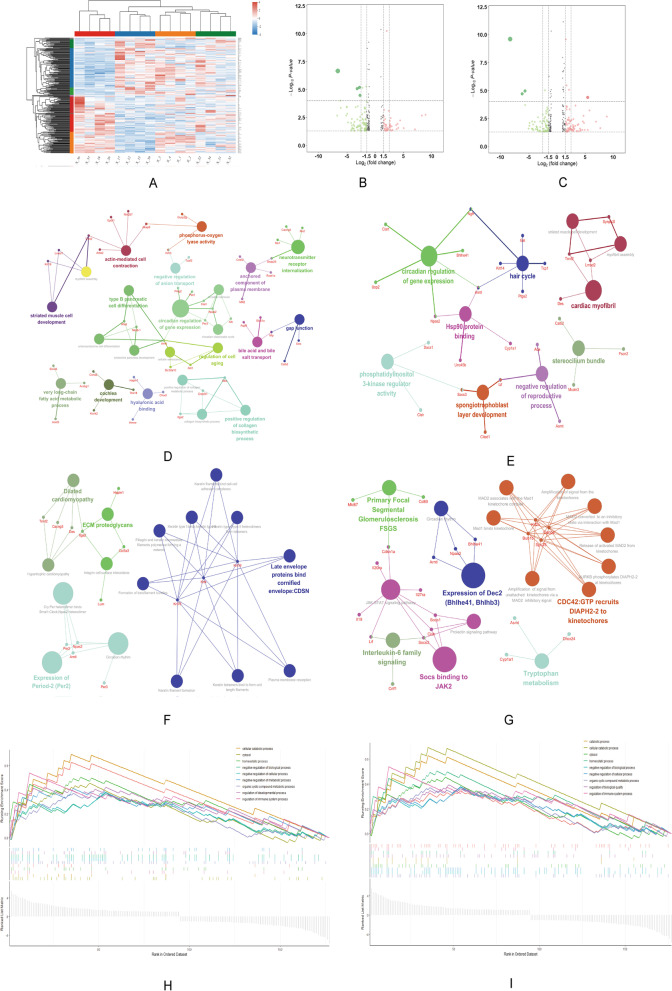
SIN affected renal mRNA alteration in DN rats. Heatmap showed the clustering of mRNAs in the renal tissues of each group. In the clustering analysis, upregulated and downregulated genes were marked in red and blue, respectively (**A**). The volcano plot compared the mRNA expression profile in renal tissues of DN vs. SIN (20 mg/kg) group and DN vs. SIN (40 mg/kg) group (**B-C**). Gene functions and pathways were analyzed by using ClueGO and CluePedia plugins in Cytoscape: The network of hub genes and GO interaction of DN vs. SIN (20 mg/kg) group and DN vs. SIN (40 mg/kg) group (**D-E**); The network of hub genes and pathways interaction of DN vs. SIN (20 mg/kg) group and DN vs. SIN (40 mg/kg) group (**F-E**). GSEA-based GO analysis-enrichment plots of representative gene sets of DN vs. SIN (20 mg/kg) group and DN vs. SIN (40 mg/kg) group (**H-I**).

We first analyzed the gene expression level in different samples, Fig. 5A showed the gene expression level in samples from DN, normal and SIN-treated groups. Then, we investigated DEGs in DN group compared with low and high dose SIN-treated DN group. We found 232 DEGs (*P* < 0.05 and fold change > 1 or less than − 1) in DN versus SIN (20 mg/kg) group and 264 DEGs in DN group versus SIN (40 mg/kg)-treated group, which were showed in volcano plots (Fig. 5B, C).

ClueGO and CluePedia were employed to identify potential roles of these DEGs in DN process, and the biological process of GO analysis was focused. The p-value < 0.05 was accepted significant. GO analysis of the DEGs between DN versus SIN (20 mg/kg) group showed that the top five biological process were very long-chain fatty acid metabolic process, neurotransmitter receptor internalization, negative regulation of anion transport, bile acid and bile salt transport, and actin-mediated cell contraction (Fig. 5D). In addition, GO analysis of the DEGs between DN versus SIN (40 mg/kg) group showed that the top five biological process were circadian regulation of gene expression, phosphatidylinositol 3-kinase regulator activity, spongiotrophoblast layer development, striated muscle cell development, and myofibril assembly (Fig. 5E).

After the analysis of pathway enrichment analysis (KEGG, REACTOME, REACTOME Reactions, and Wikipathways) of DEGs between DN versus SIN (20 mg/kg)-treated group, the five most significantly enriched pathways were integrin cell surface interactions, ECM proteoglycans, circadian rhythm, expression of Period-2, and keratin type I binds keratin type II (Fig. 5F). The top five pathways from pathway enrichment analysis of DN versus SIN (40 mg/kg) group were interleukin-6 family signaling, JAK-STAT signaling pathway, socs binding to JAK2, tryptophan metabolism, and circadian rhythm (Fig. 5G).

GO analysis of DEGs could forecast the potential effect on the cellular biological processes. But it is hard to identify the attribution of DEGs to specific GO terms. GSEA could rank all genes according to the expression level and could be employed to figure out the roles of DEGs on specific GO terms. Therefore, we used GSEA to identify critical GO terms affected by SIN. In DN versus SIN (20 mg/kg) group, the top 9 GO terms analyzed by GSEA were cytosol, cellular catabolic process, regulation of immune system process, homeostatic process, catabolic process, organic cyclic compound metabolic process, negative regulation of cellular process, negative regulation of biological process, and regulation of developmental process (Fig. 5H). In DN versus SIN (40 mg/kg) group, the top 9 GO terms were organic cyclic compound metabolic process, negative regulation of biological process, negative regulation of cellular process, cytosol, cellular catabolic process, catabolic process, regulation of biological quality, homeostatic process, and regulation of immune system process (Fig. 5I).

### Differential Metabolite Analysis of serum

**Fig. 6:**
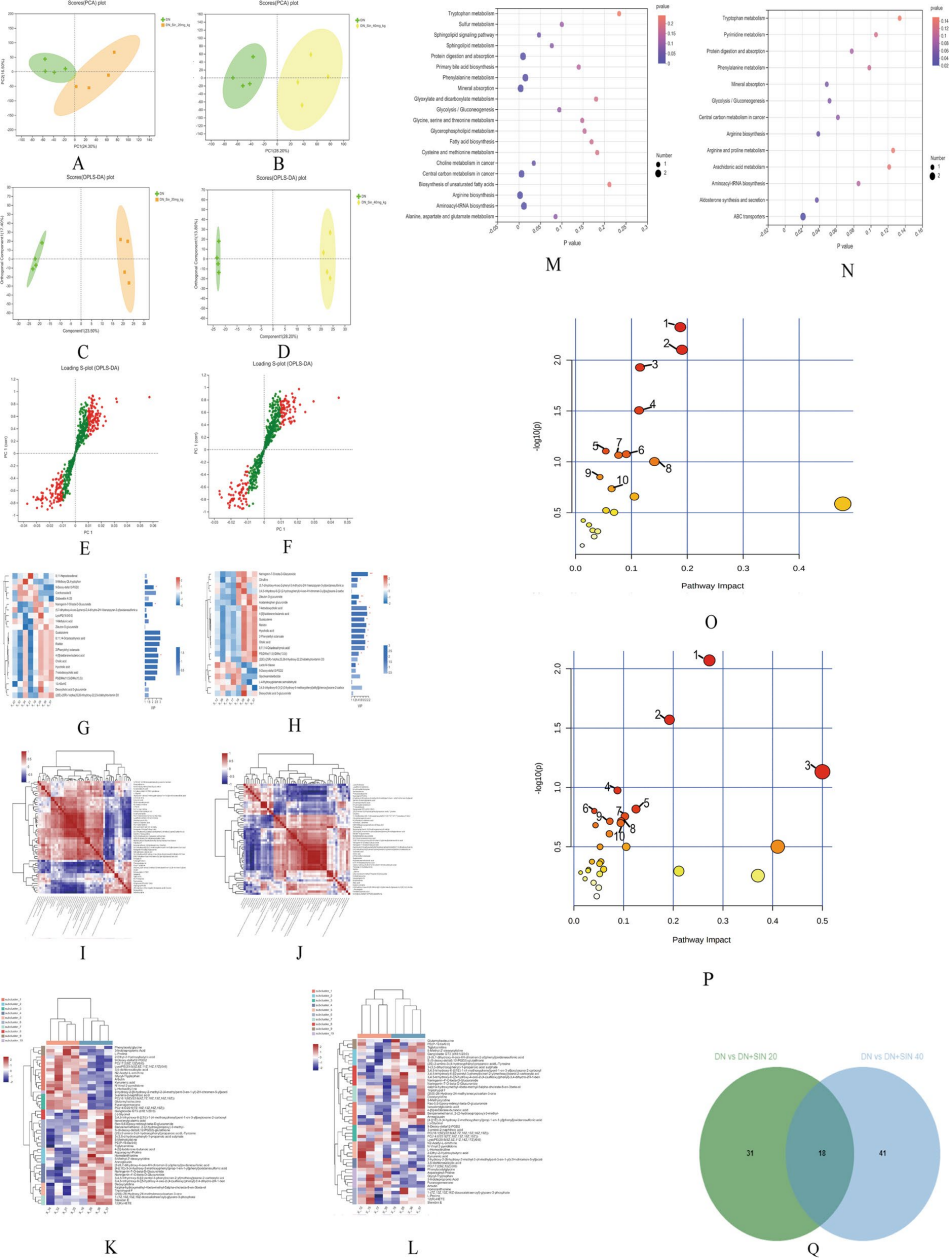
Serum metabolomics for SIN treated DN rats. PCA score plot of DN vs. SIN (20 mg/kg) group and DN vs. SIN (40 mg/kg) group (**A-B**). OPLS-DA score plot of DN vs. SIN (20 mg/kg) group and DN vs. SIN (40 mg/kg) group (**C-D**); S-plot was used to select the changes of metabolites, the variables in red were the lipids with VIP > 1.0. S-plot of DN vs. SIN (20 mg/kg) group and DN vs. SIN (40 mg/kg) group (**E-F**). VIP value analysis of DN vs. SIN (20 mg/kg) group and DN vs. SIN (40 mg/kg) group (**G-H**). Heatmap of Pearson’s correlations of DN vs. SIN (20 mg/kg) group and DN vs. SIN (40 mg/kg) group (**I-J**). Heatmap of different metabolites of DN vs. SIN (20 mg/kg) group and DN vs. SIN (40 mg/kg) group (**K-L**). KEGG enrichment analysis of different metabolites of DN vs. SIN (20 mg/kg) group and DN vs. SIN (40 mg/kg) group (**M-N**). Summary of pathway analysis with MetaboAnalyst 5.0: (1) Linoleic acid metabolism; (2) alpha-Linolenic acid metabolism; (3) Arginine biosynthesis; (4) Retinol metabolism; (5) Fatty acid elongation; (6) Arginine and proline metabolism; (7) Arachidonic acid metabolism; (8) Glycerophospholipid metabolism; (9) Phenylalanine metabolism; (10) Histidine metabolism in DN vs. SIN (20 mg/kg) group (**O**). (1) Retinol metabolism; (2) Arginine biosynthesis; (3) Biosynthesis of unsaturated fatty acids; (4) Sphingolipid metabolism; (5) Linoleic acid metabolism; (6) Aminoacyl-tRNA biosynthesis; (7) Arachidonic acid metabolism; (8) alpha-Linolenic acid metabolism; (9) Tryptophan metabolism; (10) Glycerophospholipid metabolism in DN vs. SIN (40 mg/kg) group (**P**). Venn diagram of the overlapping metabolites of DN vs. SIN (20 mg/kg) group and DN vs. SIN (40 mg/kg) group (**Q**)

In this study, we combined the positive and negative ion mode for later analyses. First, PCA was performed on serum samples and clear differences in the serum metabolome were observed between DN and SIN-treated groups (Fig. 6A, B). OPLS-DA was then conducted to identify and characterize metabolites between DN versus SIN (20 mg/kg) group and DN versus SIN (40 mg/kg) group (Fig. 6 C, D). The subsequent S-plots were applied to identify significantly changed metabolites in between DN versus SIN (20 mg/kg) group and DN versus SIN (40 mg/kg) group (Fig. 6E, F). VIP variables (generated after OPLS-DA analysis) > 1.0 were selected to later analyses. In addition, the significantly different metabolites with VIP > 1.0 in DN versus SIN (20 mg/kg) group and DN versus SIN (40 mg/kg) group were also filtered from the S-plot for later analysis, we selected the top 30 metabolites according to the VIP value between DN versus SIN (20 mg/kg) group and DN versus SIN (40 mg/kg) group (Fig. 6G, H). Pearson’s correlation analysis and hierarchical clustering analysis were applied to determine the relationships among the top 50 metabolites in DN versus SIN (20 mg/kg)-treated group and DN versus SIN (40 mg/kg)-treated group (Fig. 6I-J). The heatmaps were used to show the specific metabolic biomarkers distinguished between DN versus SIN (20 mg/kg) treatment group and DN versus SIN (40 mg/kg) treatment group (Fig. 6K, L). In addition, KEGG enrichment analysis was conducted to distinguish the different metabolites of DN vs. SIN (20 mg/kg) group and DN vs. SIN (40 mg/kg) group (Fig. 6M, N).

The metabolic biomarkers altered in the SIN (20 mg/kg) and SIN (40 mg/kg)-treated group versus DN group were identified to be involved in top 10 potential pathways based on metabolic pathway analysis with MetaboAnalyst 5.0, including (1) Linoleic acid metabolism; (2) alpha-Linolenic acid metabolism; (3) Arginine biosynthesis; (4) Retinol metabolism; (5) Fatty acid elongation; (6) Arginine and proline metabolism; (7) Arachidonic acid metabolism; (8) Glycerophospholipid metabolism; (9) Phenylalanine metabolism; (10) Histidine metabolism in SIN (20 mg/kg)-treated group versus DN group (Fig. 6O). And, (1) Retinol metabolism; (2) Arginine biosynthesis; (3) Biosynthesis of unsaturated fatty acids; (4) Sphingolipid metabolism; (5) Linoleic acid metabolism; (6) Aminoacyl-tRNA biosynthesis; (7) Arachidonic acid metabolism; (8) alpha-Linolenic acid metabolism; (9) Tryptophan metabolism; (10) Glycerophospholipid metabolism in SIN (40 mg/kg) group versus DN group (Fig. 6P). Furthermore, Venn diagram was used to determine the overlapping metabolites of DN vs. SIN (20 mg/kg) group and DN vs. SIN (40 mg/kg) group (Fig. 6Q). The 18 overlapping metabolites included 4-[5]-ladderane-butanoic acid; Asparaginyl-Proline; (-)-Glycinol; Benzenemethanol, 2-(2-hydroxypropoxy)-3-methyl-; 2-hydroxy-2-[8-hydroxy-2-methyl-2-(4-methylpent-3-en-1-yl)-2 H-chromen-5-yl]acetic acid; Arbutin; Naringenin-4’-O-beta-D-Glucuronide; Phenylacetylglycine.

S-(9-deoxy-delta9,12-PGD2)-glutathione; [3-(6,7-dihydroxy-4-oxo-4 H-chromen-2-yl)phenyl]oxidanesulfonic acid; (2 S)-2-amino-3-(4-hydroxyphenyl)propanoic acidL-Tyrosine; Naringenin-7-O-beta-D-Glucuronide; Furanogermenone; 3,5-dichlorosalicylic acid; Rac-5,6-Epoxy-retinoyl-beta-D-glucuronide; 3,4,5-trihydroxy-6-[(2-pentyl-3-phenyloxiran-2-yl)methoxy]oxane-2-carboxylic acid; Ganglioside GT3 (d18:1/20:0); N2-Acetyl-L-ornithine, which were showed in Table 2.

**Table 2:** Eighteen differential metabolites identified from different groups

Metabolite	Formula	DN vs. DN + SIN (20 mg/kg)				DN vs. DN + SIN (40 mg/kg)			
		VIP (OPLS-DA)	FC	P value	FDR	VIP (OPLS-DA)	FC	P value	FDR
4-[5]-ladderane-butanoic acid	C16H22O2	2.750310196	1.174397407	0.04862	0.3666	2.088799602	1.10438095	0.01263	0.14
Asparaginyl-Proline	C9H15N3O4	1.335358198	1.037578288	0.0385	0.3581	1.264694445	0.96283903	0.02319	0.18
(-)-Glycinol	C15H12O5	3.049088211	1.293369664	0.00309	0.269	2.642880823	1.28674699	0.02533	0.19
Benzenemethanol, 2-(2-hydroxypropoxy)-3-methyl-	C11H16O3	1.70174831	1.076959396	0.02309	0.3376	2.238934972	1.15231119	0.00647	0.11
2-hydroxy-2-[8-hydroxy-2-methyl-2-(4-methylpent-3-en-1-yl)-2 H-chromen-5-yl]acetic acid	C18H22O5	1.590928708	0.935587762	0.03168	0.3443	1.567821886	0.92626544	0.04701	0.24
Arbutin	C12H16O7	1.927342137	0.887000596	0.003632	0.2768	2.166510528	0.85513078	7.2E-05	0.02
Naringenin-4’-O-beta-D-Glucuronide	C21H20O11	1.909917025	1.10625	0.04217	0.3617	2.112485633	1.12457454	0.00862	0.13
Phenylacetylglycine	C10H11NO3	1.505632048	0.95286253	0.03071	0.3443	1.204458293	0.96577484	0.02271	0.18
S-(9-deoxy-delta9,12-PGD2)-glutathione	C30H47N3O10S	1.156293297	1.033232089	0.04581	0.3637	1.264550908	1.04123154	0.03162	0.2
[3-(6,7-dihydroxy-4-oxo-4 H-chromen-2-yl)phenyl]oxidanesulfonic acid	C15H10O8S	1.897366035	1.077025994	0.0144	0.3273	1.561911404	1.05980816	0.02146	0.17
(2 S)-2-amino-3-(4-hydroxyphenyl)propanoic acidL-Tyrosine	C9H11NO3	1.033002195	1.0220613	0.02957	0.3443	1.12525557	1.02812129	0.01837	0.16
Naringenin-7-O-beta-D-Glucuronide	C21H20O11	2.000183288	1.08987701	0.02153	0.3376	2.112485633	1.12457454	0.00862	0.13
Furanogermenone	C15H20O2	5.906354275	0.219909605	0.002447	0.2638	4.63574522	0.26733516	0.01212	0.14
3,5-dichlorosalicylic acid	C7H4Cl2O3	3.322460748	0.795840266	0.008505	0.3178	3.073359328	0.78979524	0.02436	0.18
Rac-5,6-Epoxy-retinoyl-beta-D-glucuronide	C26H36O9	3.122146738	1.349954393	0.04043	0.3612	2.760429353	1.33053641	0.04719	0.24
3,4,5-trihydroxy-6-[(2-pentyl-3-phenyloxiran-2-yl)methoxy]oxane-2-carboxylic acid	C20H28O8	2.20559935	1.098851518	0.002855	0.269	1.506658968	1.0609901	0.01941	0.17
Ganglioside GT3 (d18:1/20:0)	C83H146N4O37	2.007697829	1.072841134	0.005428	0.2806	1.261236796	1.03349738	0.02006	0.17
N2-Acetyl-L-ornithine	C7H14N2O3	1.286873738	0.972589329	0.004259	0.2777	1.039982589	0.9754541	0.02507	0.18

### Integrated analysis of metabolomics and transcriptomics

**Fig. 7:**
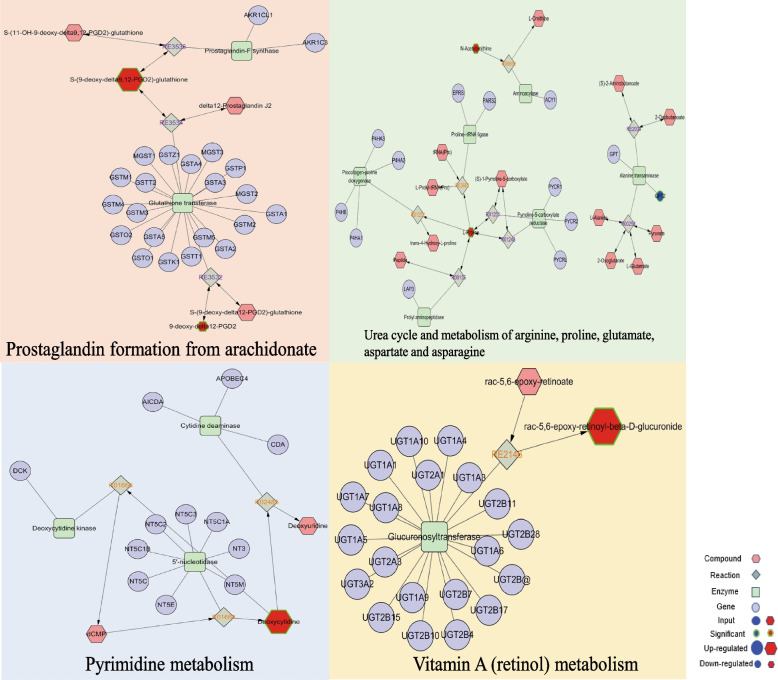
Fully connected network of differential metabolites and genes in DN versus SIN (20 mg/kg) treatment group

To obtain a comprehensive view of the mechanisms of SIN against DN, we constructed an interaction network based on metabolomics and transcriptomics. The key metabolites and putative genes in DN versus SIN (20 mg/kg) and SIN (40 mg/kg)-treated group were loaded into MetScape (a plugin in of Cytoscape), to establish a metabolite–reaction–enzyme–gene network in order to visualize the molecular mechanism of SIN in treating DN. In DN versus SIN (20 mg/kg) group, we found S-(9-deoxy-delta9,12-PGD2)-glutathione was significantly upregulated and 9-deoxy-delta12-PGD2 was significantly downregulated in prostaglandin formation from arachidonate. Deoxycytidine was significantly upregulated in pyrimidine metabolism. In addition, N-Acetylornithine, L-Proline and the expression of GPT2 were significantly downregulated in urea cycle and metabolism of arginine, proline, glutamate, aspartate and asparagine. And Rac-5,6-Epoxy-retinoyl-beta-D-glucuronide was significantly increased in vitamin A (retinol) metabolism (Fig. 7).

**Fig. 8:**
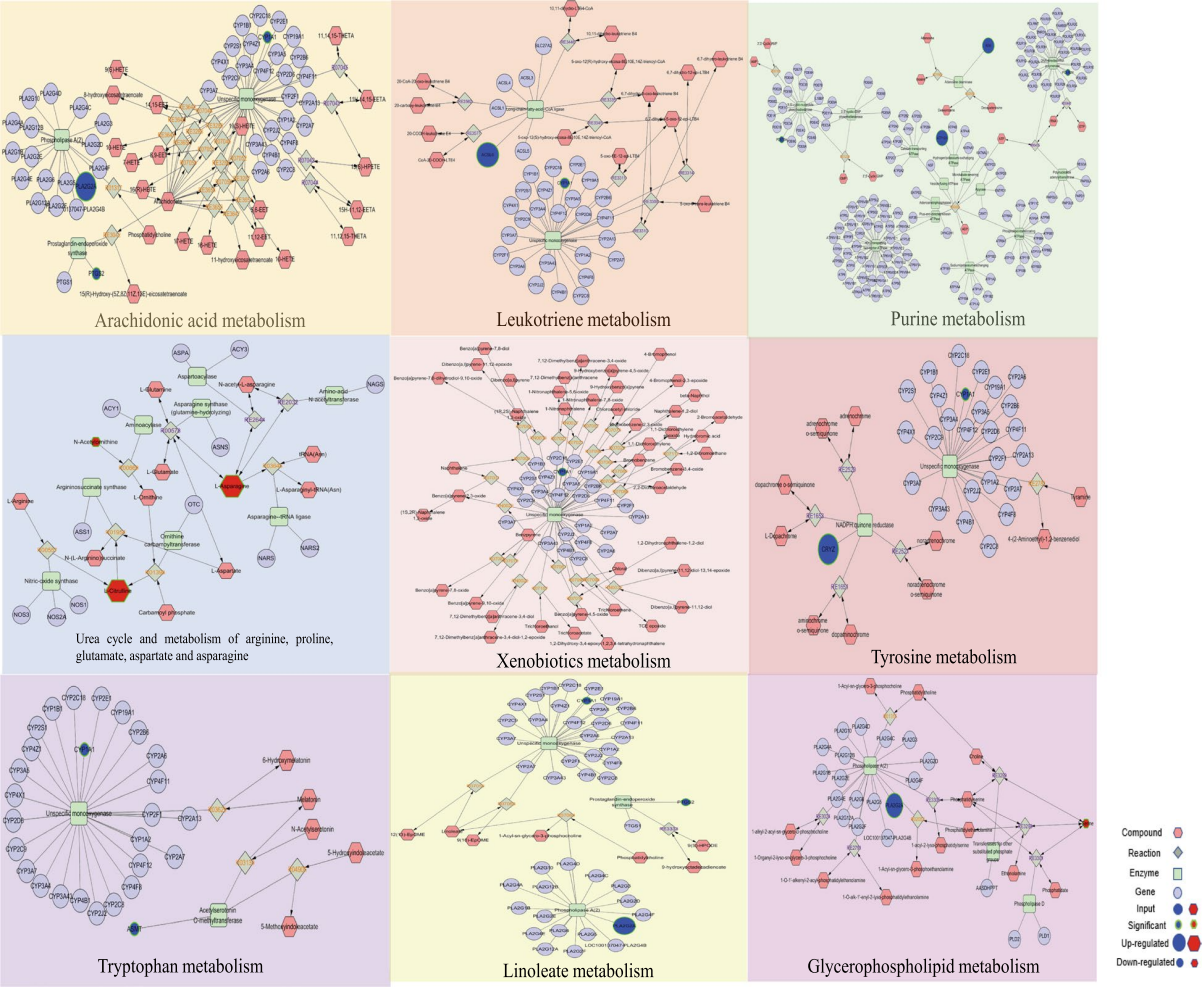
Fully connected network of differential metabolites and genes in DN versus SIN (40 mg/kg) treatment

In DN versus SIN (40 mg/kg) group, the expression level of PLA2G2A was significantly increased in arachidonic acid metabolism, while PTGS2 and CYP1A1 were significantly downregulated. PLA2G2A was upregulated and compound L-Serine was downregulated in Glycerophospholipid metabolism. In leukotriene metabolism, we found the expression of ACSL6 was significantly increased and CYP1A1 was decreased. In linoleate metabolism, PLA2G2A was significantly upregulated and PTGS2 was downregulated. In purine metabolism, the expression of ATP12A and ADA were significantly increased while the expression of POLR2H and PDE10A were significantly decreased. In addition, ASMT and CYP1A1 were significantly downregulated in tryptophan metabolism. In tyrosine metabolism, CRYZ was significantly upregulated and CYP1A1 was downregulated. Compound L-Citrulline and L-Asparagine were significantly upregulated and N-Acetylornithine was downregulated in urea cycle and metabolism of arginine, proline, glutamate, aspartate and asparagine. The expression of CYP1A1 was downregulated in xenobiotics metabolism (Fig. 8).

## Discussion

DN is a serious microvascular complication of diabetes and is the leading cause of ESRD [32]. Sinomenine has been widely applied to treat autoimmune diseases for many years. Zhang et al. found that SIN could ameliorate DOX-induced nephrotic syndrome in rats, causing the change of renal nephrin, podocin expression, hence improving the podocytes injury [33]. Qin et al. reported that SIN could effectively improve the altered expression of EMT-associated protein α-SMA, E-cadherin, fibronectin, and ECM expression that related to renal fibrosis via interrupting TGFβ/Smad3 and Wnt/β-catenin signaling [8]. SIN was also found to effectively reduce the kidney damage and inflammatory responses, balance renal oxidative stress, inhibit IkBa phosphorylation and NF-kB nuclear translocation, modulate macrophage M1/M2 polarization via an Nrf2-dependent manner. So, SIN would play a critical role in anti-inflammation and renal protection [34]. Taken together, the renal protective function of SIN has been extensively correlated with the resistance to inflammation and oxidative stress, as well as EMT progression [33–36].

Our transcriptomics results indicated that DEGs involved multiple classical biological processes, including phosphatidylinositol 3-kinase regulator activity, Hsp90 protein binding, very long-chain fatty acid metabolic process, hyaluronic acid binding, positive regulation of collagen metabolic process, positive regulation of collagen biosynthetic process. These biological processes contribute to the processes of renal fibrosis [37], disturbed glomerular endothelial stabilization [38], hyperplasia of mesangial cells [39], and inflammation [40]. In addition, the enriched pathways were found to be involved in ECM proteoglycans, circadian rhythm, interleukin-6 family signaling, JAK-STAT signaling pathway, socs binding to JAK2, and tryptophan metabolism. The enriched pathways involved in hypertension [41], renal information [42], fibrosis [41, 43, 44], and cell apoptosis [9].

Combined the results of network pharmacology and transcriptomics, we found JAK-STAT signaling pathway is a most important pathway which may be involved in the mechanisms of SIN treating DN. The JAK-STAT signaling involved in key mechanisms for several cytokines and growth factors [45]. JAK can be activated by many immune cytokines, which will in turn stimulate cellular events, such as proliferation, differentiation, migration, and apoptosis [46]. The activation of the JAK-STAT signaling has been reported to stimulate the excessive growth of glomerular mesangial cells, which lead to DN advancement [47]. STAT3 has been showed to be activated in the early stage of DN [48]. These studies indicate an important link between the JAK-STAT signaling pathway and the development of DN.

Our metabonomics results showed that SIN might improve DN via several metabolites and some metabolites have been found to be associated with the development of DN. Arbutin has anti-oxidative and anti-inflammatory activities, which could markedly improve renal function, and attenuate inflammation and cell apoptosis by modulating PI3K/Akt/Nrf2 signalling in acute kidney injury [49]. Phenilacetylglycine is a kind of fatty acid catabolites and has been reported as an early biomarker of kidney dysfunction in an animal model of ischemia/reperfusion injury [50]. Ganglioside is especially abundant in renal tissue and is known as maintaining the charge-selective filtration barrier of glomeruli. Altered expression of ganglioside was pathologically associated with glomerular hypertrophy occurring in DN kidneys [51].

Uric acid is an end product of the purine metabolism and excreted predominantly by the proximal tubules. For patients with DN, higher uric acid levels are associated with higher microalbuminuria, lower eGFR [52]; Linoleic acid would ameliorate hyperuricemia, insulin resistance and renal inflammation, accompanied with the downregulation of renal GLUT9 and URAT1 in fructose-fed rats and the inhibition of NLRP3 inflammasome and TLR4/MyD88 signaling [53]; Tyrosine metabolism is correlated with multiple diseases such as fatty liver, insulin resistance, and obesity [54]. Nitrotyrosine has been reported to participate in the progression of diabetes and its complications, the increased levels of nitrotyrosine can affect renal pathology and lead to renal dysfunction in diabetic rats [55]. Elevated levels of nitrotyrosine is positively correlated with patients with DN [56]. Nitrotyrosine has been found to induce glomerular mesangial cells to express NF-κB, MCP-1, and TGF-β1, leading to inflammation and aggravating nephropathy [57]; Recent study has confirmed that the increment of leukotriene in the kidneys after ischemia, which could further mediate multiple inflammatory reactions causing the kidney damage [58]. The role of leukotriene in glomerular injury has been confirmed, increased recruitment or activation of polymorphonuclear cells and elevated level of leukotriene B4 in the kidney would eventually decrease the glomerular filtration rate [59]; Glycerophospholipids are the major part of the cell membranes and involved in cell signaling, membrane anchoring and substrate transport. One study has discovered that abnormal glycerophospholipids were associated with patients and animals with CKD [60].

## Conclusion

In our study, we firstly used a novel combined strategy to identify the potential targets and mechanisms of SIN administration in treating DN according to metabolomics, transcriptomics, histology, biochemical parameters and network pharmacology. It will provide a new paradigm to figure out the potential mechanisms of pharmacological effects of a natural compound. In addition, our research offered information and theoretical foundation for an in-depth observation of mechanisms and provided the support for SIN clinical practice. Future systematic molecular biology experiments will be needed to confirm the precise mechanism.

## Data Availability

All raw next-generation sequencing data has been deposited in GEO database with the accession number GSE200221.
